# Torsades de pointes in the PACU after outpatient endoscopy: a case report

**DOI:** 10.1186/s12871-021-01457-9

**Published:** 2021-12-01

**Authors:** Andrew Schaar, Mark Liu, Michael Patzkowski

**Affiliations:** Brooke Army Medical Center – Fort Sam Houston, Anesthesiology Department, 3351 Roger Brooke Drive, San Antonio, TX 78234 USA

**Keywords:** Hypokalemia, Torsades de pointes, Acquired long QT syndrome, Congenital long QT syndrome, Magnesium

## Abstract

**Background:**

This case demonstrates the severe electrolyte derangements that may present after a common therapy such as a bowel preparation for an outpatient procedure and the rare yet potential detrimental outcomes of those abnormalities. It also highlights the implications of long QT syndrome regarding pharmacology and treatment.

**Case presentation:**

We present a case of 48 year-old female with severe electrolyte derangements and long QT syndrome (LQTS) leading to Torsades de Pointes (TdP), pulseless ventricular fibrillation, and unsynchronized defibrillation in the post anesthesia care unit (PACU) after uneventful upper and lower endoscopy. This led to an unanticipated intensive care unit admission for aggressive electrolyte repletion, cardiology consultation, and implantable cardioverter defibrillator (ICD) placement.

**Conclusions:**

This is a rare presentation after an outpatient procedure that would have had a detrimental outcome if not promptly diagnosed and treated appropriately. Therefore, we aim to provide further insight into the diagnosis and treatment of severe hypokalemia and long QT syndrome resulting in Torsades de Pointes and ventricular fibrillation.

## Background

Profound hypokalemia and long QT syndrome are rare yet potentially life threatening abnormalities. They become even more significant when discovered postoperatively in a patient after a common outpatient procedure such as an endoscopy. Severe hypokalemia requires a high index of suspicion for diagnosis and prompt repletion to avoid further sequelae whereas long QT syndrome may be congenital or acquired and may lead to sudden cardiac death. Written HIPAA consent has been obtained from the patient and is available for review.

## Case presentation

### Patient information

A 48 year-old female with diarrhea predominant irritable bowel syndrome and gastroesophageal reflux disease is scheduled for esophagogastroduodenoscopy and colonoscopy under monitored anesthesia care. Her other past medical history is significant for hypertension, depression, anxiety, and a history of “paroxysmal supraventricular tachycardia (PSVT)”. Regarding the diagnosis of PSVT, she described self-resolving syncopal episodes in her early adult life. She stated she was currently being evaluated by a cardiologist with the suspected diagnosis of PSVT yet no diagnostic tests had been performed prior to her scheduled procedure. Pertinent medications include fluoxetine, buproprion, alprazolam, diltiazem, amlodipine, omeprazole, and ondansetron. Social and family history were non-contributory. She completed a bowel preparation the day prior to presentation as instructed along with minimal oral intake in the preceding days due to her persistent nausea.

### Clinical findings

The anesthesia team was contacted by the patient’s nurse in the post anesthesia care unit (PACU) for persistent postoperative nausea, a frequent issue encountered in the PACU. The patient’s physical exam was unremarkable with normal vital signs. Electrocardiogram (EKG) on the bedside monitor appeared normal. The patient appeared mildly uncomfortable stating nausea slightly worse than her recent baseline.

### Timeline

The patient had an uneventful intraoperative course under monitored anesthesia care with 2 mg (mg) of midazolam given preoperatively and propofol titrated intraoperatively to desired sedation level. Postoperatively, she was given intravenous (IV) ondansetron 4 mg and IV promethazine 12.5 mg for persistent nausea as part of PACU orders placed by the intraoperative anesthesia provider. The PACU anesthesia provider along with gastroenterology were informed after nausea persisted beyond these measures. A scopolamine patch was ordered by her surgical team as the patient was scheduled to be discharged home and a scopolamine patch would potentially provide prolonged antiemetic benefits. Her nausea persisted with now heightened anxiety. Twenty milligrams of propofol was given under full monitors with no effect noted. Her anxiety and restlessness increased for which her home oral alprazolam 0.5 mg was given.

Approximately 60 minutes later, the patient’s bedside nurse alerted the PACU anesthesia team that the patient appeared to have a 10 second run of presumed supraventricular tachycardia for which she had a history of. A stat EKG was ordered yet prior to being completed, the patient went unconscious and into pulseless ventricular fibrillation. Unsynchronized defibrillation at 200 joules was performed followed by advanced cardiovascular life support. Approximately 5 chest compressions were delivered before the patient was noted to be awake and moving spontaneously. No medications were given during ACLS. Return of spontaneous circulation obtained after approximately 30 s from the time she was noted to be unconscious and the patient was now awake and following commands.

### Diagnostic assessment

**Fig. 1:**
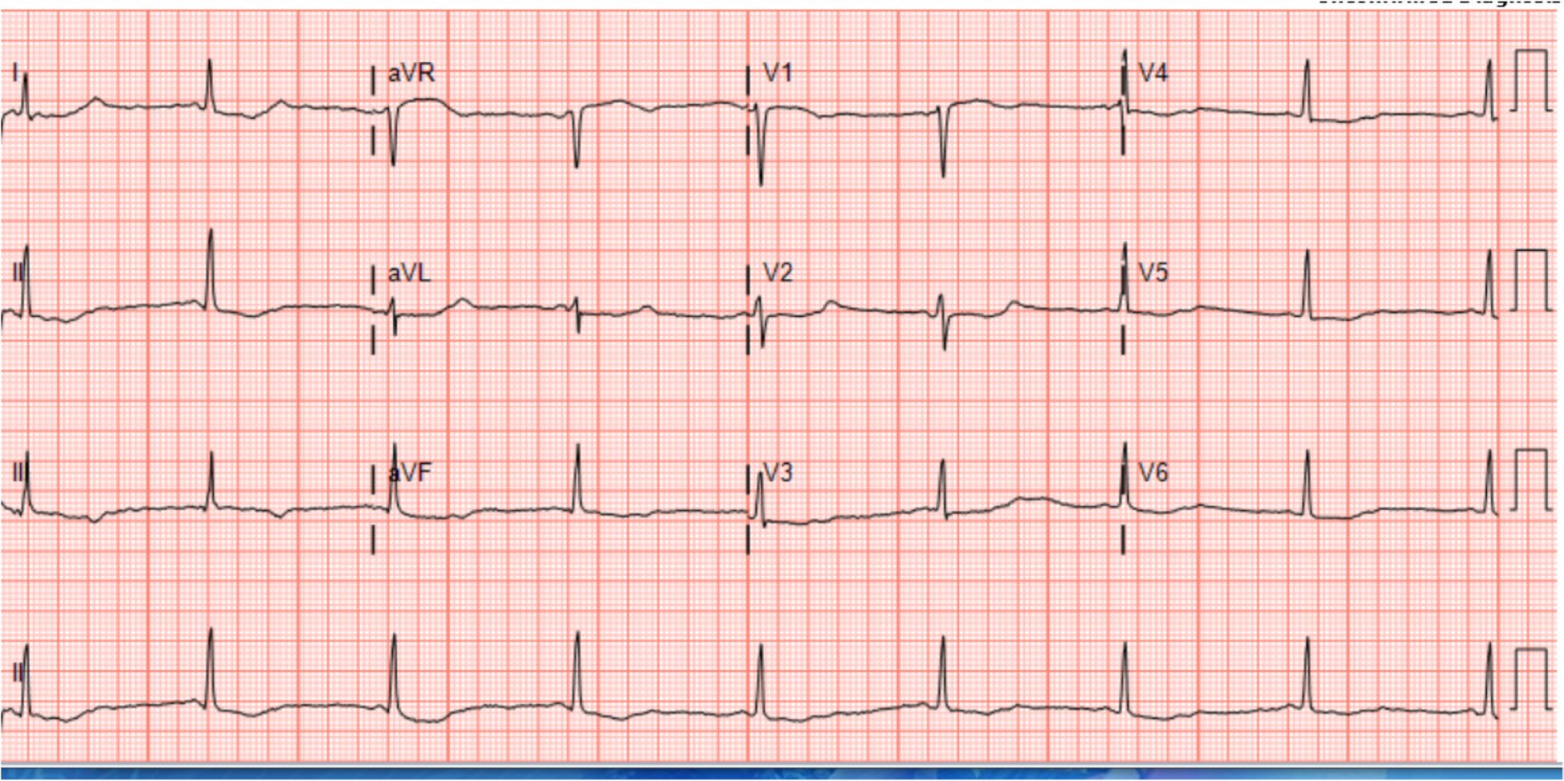
Post cardiac arrest EKG demonstrating inverted T waves, U waves, and QTc >600msec

**Fig. 2:**
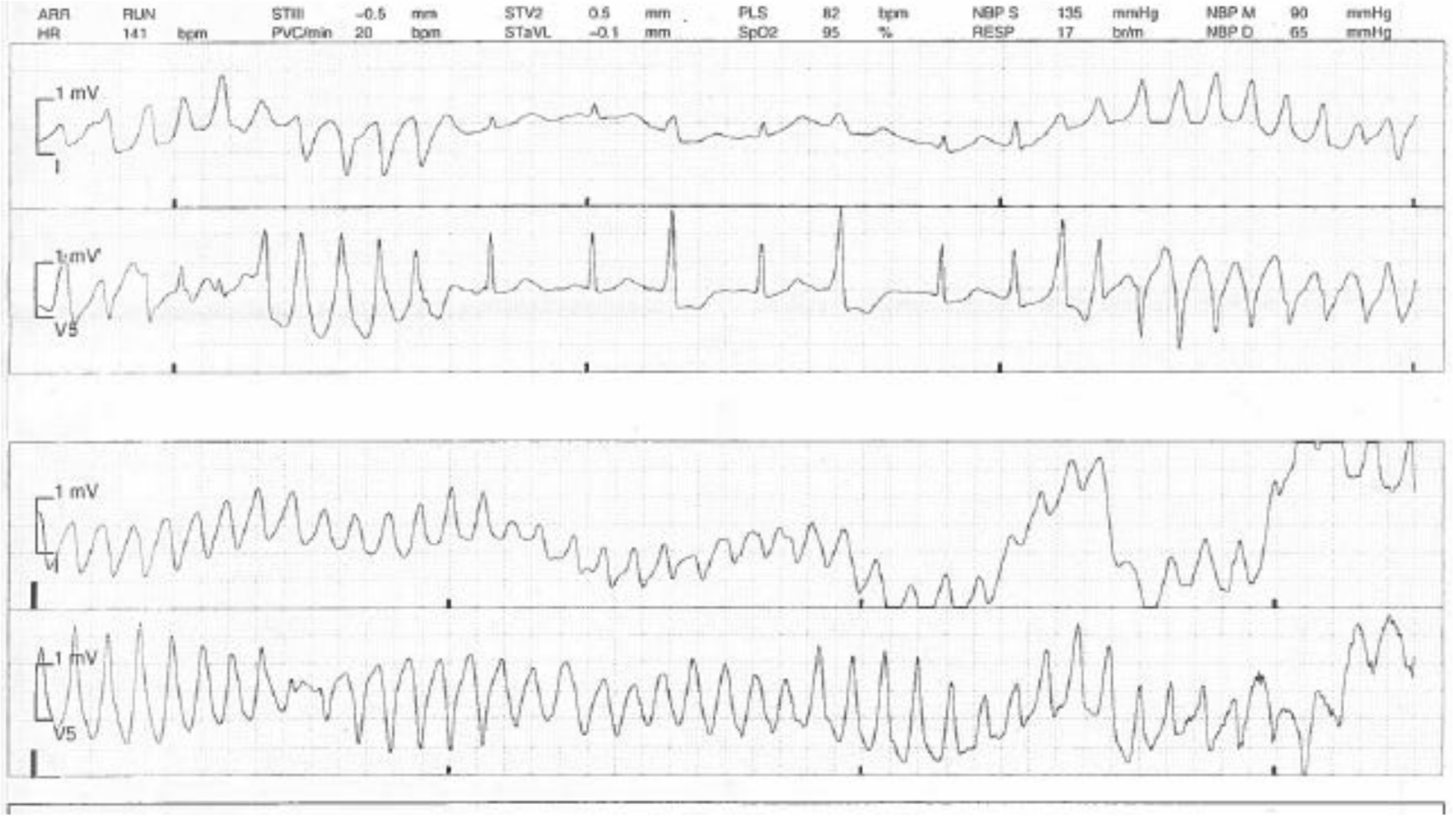
Torsades de Pointes in the PACU

Stat EKG revealed inverted T waves, prominent U waves, and a prolonged QTc of > 600 milliseconds (Fig. 1). The rhythm strip from her earlier episode of presumed SVT was able to be retrieved (Fig. 2) and was consistent with polymorphic ventricular tachycardia (Torsades de Pointes). Two grams of IV magnesium was initiated. Laboratory evaluation revealed a potassium level of 2.4 mmol/L (critically low; Normal 3.5–4.5), phosphorus of 1.0 mg/dL (low; Normal 0.97–1.45), ionized calcium 1.04 mmol/L (low; Normal 1.2–1.32), magnesium of 2.3 mg/dL (normal 1.6–2.4), glucose 144 mg/dL (normal fasting level 60–100). Chest radiograph obtained and was normal. The diagnoses of severe hypokalemia, hypophosphatemia, hypocalcemia, and suspected acquired long QT syndrome were made. The prognosis of those conditions is favorable with electrolyte repletion and avoidance of QT prolonging medications.

### Therapeutic intervention

After the diagnosis of Torsades de Pointes, 2 g of IV magnesium was initiated and infused over 15 min. Aggressive potassium and phosphorus repletion was initiated with both IV and oral replacement as the patient was awake, fully oriented, and following commands. The medical intensive care unit was contacted and admitted the patient for continued electrolyte repletion and further workup.

### Follow-up and outcomes

**Fig. 3:**
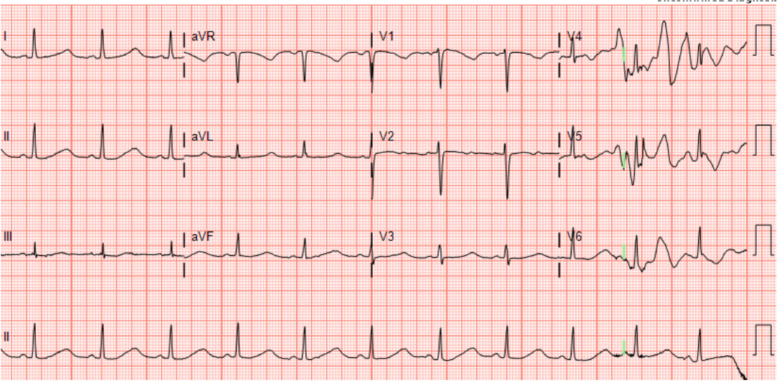
EKG in CCU capturing recurrent Torsades de Pointes

During her hospital stay, the patient continued to have long QT syndrome and experienced two additional episodes of recurrent Torsades de Pointes (Fig. 3), pulseless ventricular fibrillation, and cardiac arrest requiring defibrillation. These episodes occurred in the intensive care unit despite discontinuation of all QT prolonging medications and a normal electrolyte panel. Each episode responded to defibrillation with return to normal sinus rhythm. Interventional cardiology was consulted for further evaluation and treatment and she was transferred to the Cardiac Critical Care Unit. After failure of medical management and persistent prolonged QT syndrome, the diagnosis of congenital long QT syndrome was made. This was acutely exacerbated by secretory diarrhea, electrolyte abnormalities, and QT prolonging medications. An implantable cardioverter defibrillator was placed on day 8 of her hospital stay and she was discharged home on hospital day 10.

## Discussion & Conclusions

**Fig. 4:**
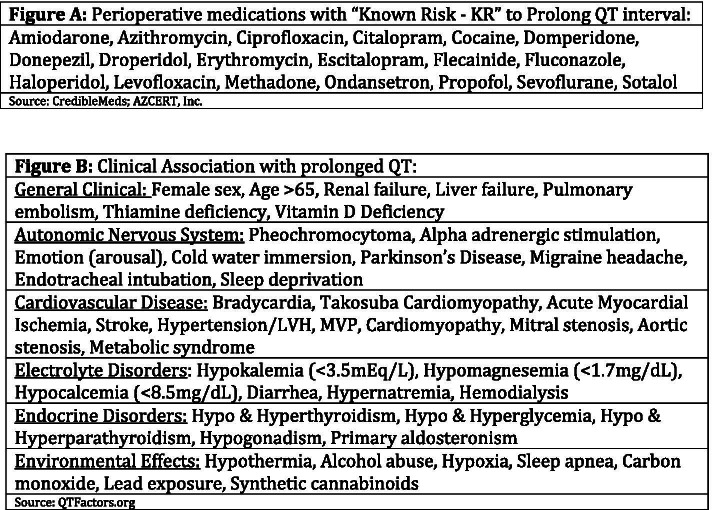
**A** Perioperative Medications with Known Risk for QT prolongation.  **B** Risk factors for prolonged QTc and TdP

This case describes a rare presentation after a common outpatient procedure and a common postoperative complaint. The case highlights the substantial electrolyte derangements that may be seen with persistent secretory diarrhea. It also highlights the importance of prompt recognition of long QT syndrome as if unrecognized, may result in sudden cardiac death. Regarding the incidence of TdP, Nuttall et al. [1] investigated the significance of the black box warning on the previously often used antiemetic, Droperidol. Only 3 cases of TdP were discovered in over 200,000 surgical cases over a 6-year period. While congenital LQTS is estimated to have a prevalence of 1:3000–7000, the presentation of TdP in the perioperative setting is exceedingly rare. Anesthesia providers frequently administer medications known to be associated with QT prolongation (Fig. 4A) [2]. Most sources agree the QTc interval is considered to be prolonged if > 450 ms for males and > 470 ms for females [3, 4]. It is imperative to recognize the risk factors for QT prolongation and TdP (Fig. 4B) [5] and to limit iatrogenic factors as much as possible to avoid potential negative outcomes.

The relevant medical literature includes several case reports of QT interval prolongation either acquired or congenital, descriptions of the pathophysiology underlying the condition, and monitoring and treatment guidelines for clinicians. Mouyis et al. [6] describes a case of an 81-year-old female with a 3-year history of noninfective diarrhea and recurrent syncopal events over the preceding 3 months. She was admitted to the cardiology service where telemetry and holter analysis confirmed multiple episodes of Torsade de Pointes. She was eventually discharged after ICD placement. The authors concluded that chronic diarrhea can result in life threatening polymorphic VT due to hypokalemia and QTc prolongation and that ICD placement should be considered in these patients. Trinkley et al. [7] lists several risk factors for drug induced TdP including hypokalemia, female sex, drug-drug interactions, advancing age, genetic predisposition, hypomagnesemia, heart failure, bradycardia, and corrected QT interval prolongation. See Fig. 4B for a more comprehensive list of risk factors. Coleman et al. [8] describes a case of TdP in a child undergoing an outpatient procedure (myringotomy). The patient had known congenital LQTS with an AICD in place. The AICD failed perioperatively resulting in TdP requiring external defibrillation. Thomas et al. [9] describes TdP as typically occurring in self-limiting bursts, causing dizziness and syncope, yet may occasionally progress to ventricular fibrillation and sudden death. Furthermore, management of TdP includes removal or correction of precipitants, including discontinuation of culprit drugs and institution of cardiac monitoring. Electrolyte abnormalities and hypoxia should be corrected with potassium concentrations maintained in the high normal range. Immediate treatment is by intravenous administration of magnesium sulfate, terminating prolonged episodes using electrical cardioversion, and expert consultation. There are numerous forms of congenital long QT syndrome and the classification/ pathophysiology of these conditions is available to review in other resources.

The number of known medications with QT prolonging potential is well over 200 (AZCERT, Inc.). A reference to many, yet not all, commonly used perioperative medications with a known risk (KR) of TdP is available in Fig. 4A. It is important to note that this patient had several risk factors predisposing her to this episode of TdP including marked electrolyte derangements, the administration of more than one QT prolonging medication, female gender, and her undiagnosed congenital conduction abnormalities. It is also imperative to recognize that her history of presumed “paroxysmal SVT” should have prompted further questioning and investigation. It is most likely that her history of previous syncopal episodes were episodes of TdP that she miraculously survived without intervention. The history of syncope attributed to an arrhythmia (albeit undiagnosed) may represent a life-threatening condition and appropriate evaluation should be performed to avoid potential catastrophic outcomes.

The primary lesson of this case report includes having a high index of suspicion for electrolyte derangements after chronic diarrhea complicated by bowel preparation and minimal oral intake in the preceding days. Additionally, it highlights the importance maintaining a broad differential diagnosis and considering uncommon conditions when evaluating patients with seemingly common presentations such as post-operative nausea and vomiting. In conclusion, we believe it would be reasonable to consider pre-operative laboratory and/or electrocardiogram in patients with known or suspected cardiac conduction abnormalities and those with a history of prolonged secretory diarrhea given the potential for marked electrolyte derangements.

### Patient perspective

N/A.

### Informed consent

Obtained; available on file.

## Data Availability

All data generated or analyzed during this study are included in this published article.

## Abbreviations

TdP: Torsades de Pointes
PSVT: Paroxysmal Supraventricular Tachycardia
PACU: Post anesthesia care unit
EKG: Electrocardiogram
mg: Milligrams
IV: Intravenous
